# Research progress in *SYNGAP1*-related neurodevelopmental disorders: from pathogenesis to therapeutic strategies

**DOI:** 10.3389/fneur.2026.1773363

**Published:** 2026-02-12

**Authors:** Jia Zhang, Gong xue, Xiaoqian Wang, Xueyi Rao, Jun Chen, Lijuan Fan, Liqin Liu, Jing Gan

**Affiliations:** 1 Department of Pediatrics, West China Second University Hospital, Sichuan University, Chengdu, Sichuan, China; 2 Key Laboratory of Birth Defects and Related Diseases of Women and Children (Sichuan University), Ministry of Education, Chengdu, Sichuan, China; 3 Department of Pediatrics, WCSUH-Tianfu·Sichuan Provincial Children's Hospital, Meishan, Sichuan, China

**Keywords:** antisense oligonucleotide, epilepsy, gene therapy, neurodevelopmental disorders, synaptic plasticity, *SYNGAP1*

## Abstract

*SYNGAP1*-related neurodevelopmental disorder (SRD) is a monogenic inherited brain disorder caused by heterozygous loss-of-function mutations in the *SYNGAP1* gene. The clinical presentation is complex, with core features including global developmental delay/intellectual disability, epilepsy, autism spectrum disorder, and various behavioral abnormalities. The SynGAP protein, encoded by the *SYNGAP1* gene, is a key regulatory protein in the postsynaptic density of excitatory neurons. Through its GTPase-activating protein activity and structural scaffolding functions, it plays a central role in regulating the Ras/Rap signaling pathways, AMPA receptor trafficking, and maintaining the excitatory/inhibitory balance of neural networks. Haploinsufficiency of SynGAP leads to synaptic plasticity disruption and neural circuit imbalance, thereby triggering a series of neurophysiological and behavioral phenotypes. This article systematically reviews the molecular pathogenesis of SRDs, summarizes advances in treatment from conventional anti-seizure medications to emerging precision therapeutic strategies such as gene supplementation, antisense oligonucleotide-mediated splicing modulation, and translation-activating RNAs, and discusses current research challenges and future directions. Key concepts central to understanding SRDs include the critical developmental periods during which SynGAP exerts its primary influence on synaptic maturation, and cell-type specificity, referring to the differential expression and function of SynGAP in distinct neuronal populations (e.g., excitatory pyramidal neurons vs. parvalbumin-positive interneurons), which underlies circuit-level dysfunction. The aim is to provide a comprehensive perspective for an in-depth understanding of the disease and to support the development of effective therapies.

## 1 Introduction

*SYNGAP1-*related neurodevelopmental disorders (SRDs) are rare autosomal dominant disorders, accounting for approximately 0.7–1% of all intellectual disability cases (1). Although only over a thousand cases have been confirmed worldwide to date, the detection rate in high-risk populations suggests that the true prevalence is likely substantially higher. The clinical manifestations are highly heterogeneous; however, core symptoms are highly consistent, including moderate to severe intellectual disability (ID), global developmental delay (often preceding epilepsy), a high incidence of epilepsy (up to 98%), autism spectrum disorder (ASD, about 50%), as well as motor impairments, sleep problems, behavioral abnormalities, and high pain threshold (2–5). Since *SYNGAP1* was first identified as a causative gene for non-syndromic intellectual disability in 2009, significant progress has been made in research on its molecular mechanisms and treatment strategies. This review aims to integrate the latest findings from basic research and clinical studies to provide a comprehensive overview of the pathogenesis and therapeutic prospects of SRDs.

## 2 Literature search strategy

This narrative review was conducted based on a literature search in PubMed databases, covering publications from January 2013 to October 2025. Search terms included combinations of “SYNGAP1,” “SynGAP,” “neurodevelopmental disorder,” “intellectual disability,” “epilepsy,” “pathogenesis,” and “treatment.” Inclusion criteria prioritized original research articles and high-impact reviews focused on the molecular mechanisms, clinical phenotypes, and therapeutic strategies of SYNGAP1-related disorders. Conference abstracts and non-English publications were excluded. While not a systematic review with formal risk-of-bias assessment, we endeavored to cite studies from peer-reviewed journals and to present a balanced view that includes both supportive and inconclusive findings, particularly noting the preclinical nature of many therapeutic approaches discussed.

## 3 Molecular structure and function of *SYNGAP1*

### 3.1 Gene and protein structure

**Figure 1:**
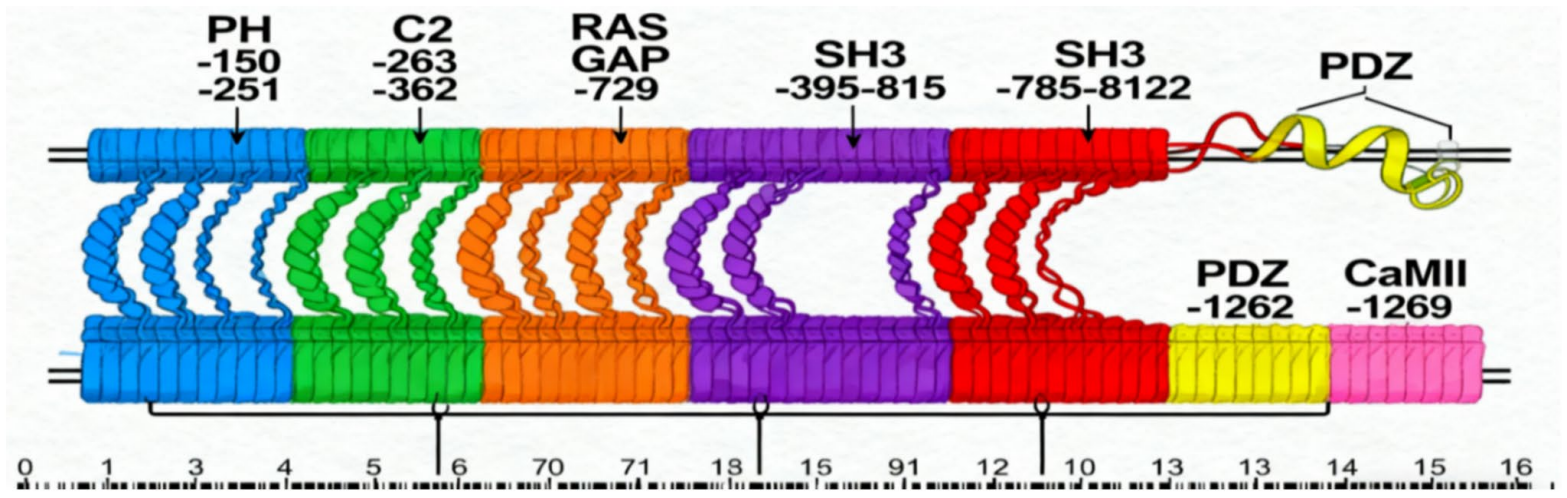
Map of SynGAP protein domains.

The *SYNGAP1* gene is located on chromosome 6p21.3 and encodes the SynGAP protein, which is enriched in the postsynaptic density (PSD) of excitatory glutamatergic neurons (6). SynGAP is a multi-domain protein. Its core functional domains consist of an N-terminal Pleckstrin Homology (PH) domain, a Protein Kinase C conserved domain 2 (C2 domain), a central Ras GTPase-activating protein (RasGAP) domain, as well as C-terminal SH3 domains, coiled-coil regions, and a PDZ-binding motif (7) (Figure 1). Together, these domains enable SynGAP to regulate both signal transduction and protein–protein interactions.

### 3.2 Alternative splicing and protein isoforms

*SYNGAP1* undergoes complex alternative splicing to produce multiple protein isoforms, with main differences in the N-terminal (e.g., A, B, C, D) and C-terminal (e.g., *α*1, *α*2, *β*, *γ*) regions. These isoforms exhibit significant spatiotemporal expression specificity and differential subcellular localization (8). For example, the *α*1 isoform is significantly upregulated in the cortex and hippocampus during late postnatal development and remains enriched in the PSD; the *α*2 isoform translocates from the cytoplasm to the PSD during development; while the *β* isoform is primarily localized to non-synaptic cytoplasmic regions. This molecular diversity suggests that different isoforms may perform specific neural functions and contributes to the phenotypic heterogeneity observed in SYNGAP1-related disorders.

The developmental expression and synaptic localization of SynGAP isoforms are highly specific: *β* is highly expressed early in development, while *α*1 increases during maturation. At the synapse, *α*1 is enriched in the PSD via its PDZ-binding motif and regulates synaptic plasticity, *α*2 shows partial PSD localization, and *β* is predominantly cytoplasmic. This molecular diversity underlies the clinical heterogeneity of SYNGAP1-related disorders: mutations may differentially affect distinct isoforms, disrupting neural circuits and signaling pathways at specific developmental stages. Consequently, isoform-specific dysfunction—such as impaired *α*1 potentially contributing to persistent cognitive deficits and epilepsy, and altered *α*2/*β* possibly affecting early neurodevelopmental milestones—may drive diverse phenotypes including intellectual disability, autism, and epilepsy (9–12). However, direct causal links between individual isoform loss and precise clinical symptoms remain to be firmly established and represent an important direction for future research.

### 3.3 *SYNGAP1* gene and its protein function

The *SYNGAP1* gene encodes a specific Ras GTPase-activating protein (RasGAP), SynGAP, which is primarily located in the postsynaptic density of glutamatergic neurons and acts as a negative regulator of excitatory synaptic strength. Pathogenic variants in this gene are mainly clustered within exons 3 to 17, following an autosomal dominant inheritance pattern. The encoded SynGAP protein localizes to dendritic spines and interacts with key synaptic proteins such as PSD-95 and CaMKII, playing a crucial role in regulating synaptic plasticity and neuronal excitability (9).

In terms of subcellular localization, SynGAP is predominantly found in the dendritic spines of neocortical pyramidal neurons and participates in the NMDA receptor (NMDAR)-dependent Ras signaling pathway. Its activity is regulated by upstream NMDARs; glutamate-induced NMDAR activation leads to CaMKII phosphorylation, which in turn activates SynGAP to modulate downstream signaling pathways.

Functionally, SynGAP affects the endocytosis of AMPA receptors and their trafficking to the postsynaptic membrane by regulating the activities of Ras and Rap This regulation leads to a reduced expression of AMPA receptors on the postsynaptic membrane and serves as a key mechanism for negatively regulating neuronal excitability. Simultaneously, SynGAP can inhibit the activity of Extracellular Signal-Regulated Kinase (ERK), thereby participating in the regulation of the cell cycle and gene transcription, which is crucial for neurodevelopment and glutamatergic neurotransmission (13–15). During the critical period of synaptogenesis, *Syngap1* is highly expressed in transgenic animal models, with its spatial distribution confined to the forebrain, particularly the hippocampal region (16), underscoring the spatiotemporally specific role of SynGAP in synaptic development.

Dysfunction of SynGAP impairs synaptic function through multiple mechanisms. *SYNGAP1* haploinsufficiency leads to cortical hyperexcitability and imbalance, including an imbalance in excitatory/inhibitory processes, which is considered a key pathophysiological mechanism for the associated clinical neurological phenotypes. Specifically, loss of function leads to accelerated maturation of glutamatergic synapses, abnormal MAP kinase signaling pathways, and impaired development of cortical neurons, and reduced synaptic plasticity. Recent studies also indicate that *SYNGAP1* haploinsufficiency affects the formation and activity of GABAergic synapses, further exacerbating functional disruption in neural networks (17). Furthermore, *SYNGAP1* mutations can lead to an increase in Ras-dependent signaling pathway proteins (including TRPV1), thereby promoting excitatory/Inhibitory (E/I) imbalance and facilitating epileptogenesis, a consequence closely tied to its central role in synaptic signal transduction (18).

### 3.4 Potential mechanistic diversity of different *SYNGAP1* variant types

While haploinsufficiency due to loss-of-function alleles is the predominant and well-established mechanism in SRDs, emerging evidence suggests that different variant types may contribute to the disease through partially divergent molecular pathways. The majority of pathogenic variants are truncating (nonsense or frameshift), leading to mRNA decay via nonsense-mediated decay (NMD) or production of unstable, nonfunctional protein fragments, resulting in pure haploinsufficiency (7, 19). In contrast, the pathogenic potential of missense variants is more complex. Some missense mutations, particularly those within the critical GAP domain or the C-terminal PDZ-binding motif, may not only cause a loss of the mutated protein’s function but could also exert a dominant-negative effect by interfering with the function of the wild-type allele, for example, through aberrant interactions within the PSD scaffold or disruption of liquid–liquid phase separation (19). Furthermore, splice-site variants can lead to aberrant splicing, potentially producing isoforms with altered functions, stabilities, or subcellular localizations, rather than simple null alleles (17). Although detailed functional characterization of many variants is still lacking, understanding these potential mechanistic nuances is crucial for explaining phenotypic variability and for future personalized therapeutic approaches, as strategies like gene supplementation or ASO-mediated splicing correction might have differential efficacy depending on the underlying molecular lesion (20, 21).

## 4 Molecular mechanisms and functional regulation of *SYNGAP1*

### 4.1 Core molecular mechanisms: synergy and separation of GAP activity and pathogenesis

The core pathogenic mechanism of SynGAP-related disorders lies in haploinsufficiency, with its function primarily manifested in two aspects that are both independent and synergistic: GAP enzymatic activity and structural scaffolding.

**Figure 2:**
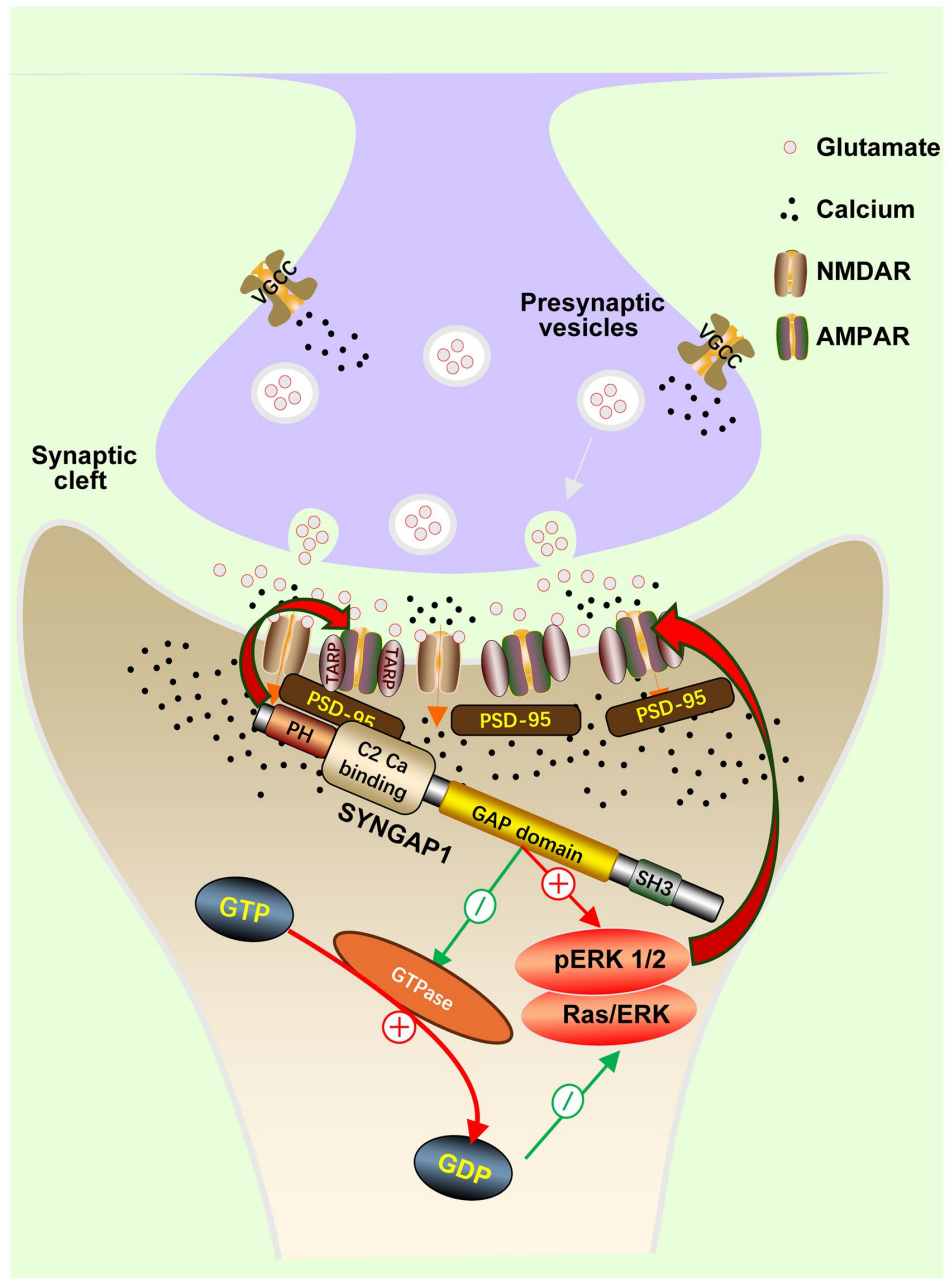
Molecular mechanisms of *SYNGAP1.*

GAP Enzymatic Activity: SynGAP, via its RasGAP domain, negatively regulates the activity of small GTPases such as Ras and Rap, promoting the hydrolysis of GTP to GDP, thereby inhibiting the downstream Ras–ERK signaling pathway (22). The GAP functional domain of *SYNGAP1* is essential for normal physiological function, and the absence of this domain leads to dysfunction (23). In SRDs, the loss of this function results in abnormally elevated ERK1/2 phosphorylation, leading to dysregulated synaptic protein synthesis, excessive insertion of AMPARs into the postsynaptic membrane, and ultimately disrupting synaptic plasticity and neuronal intrinsic excitability (19, 24) (Figure 2). Studies show that selectively disrupting GAP activity (e.g., in GAP mutant mice) leads to reduced excitability in cortical pyramidal neurons but is insufficient to induce epilepsy, suggesting that GAP activity primarily regulates the basic electrophysiological properties of neurons (25). Additionally, GAP activity may also be involved in neuronal migration, as observed migration defects in upper-layer neurons in GAP-AL mutant mice suggest its role in cellular processes (26).

Structural Scaffolding: Through its C-terminal PDZ-binding domain, SynGAP interacts with PSD-95, participating in the assembly of the PSD and liquid–liquid phase separation (LLPS). It competes with the AMPAR auxiliary subunit TARP for binding to PSD-95, thereby dynamically regulating synaptic AMPAR number and synaptic strength (27). Notably, this function is independent of its GAP activity and is crucial for the induction of long-term potentiation (LTP) and the generation of epilepsy susceptibility (Figure 2). *Syngap1* knockout mice exhibit LTP deficits and increased seizure susceptibility, whereas mice only lacking GAP activity have normal LTP, indicating that loss of structural function directly leads to the epileptic phenotype (10, 25).

### 4.2 Signaling pathway disruption

SynGAP loss of function directly leads to overactivation of the Ras–ERK signaling pathway. In *Syngap1^+/−^* mouse models, baseline Ras activity is elevated, hindering the induction of LTP by subsequent stimuli. This signaling disruption affects AMPAR trafficking, leading to premature synaptic maturation and functional imbalance, manifested as increased dendritic spine volume and a higher proportion of mushroom-shaped spines (28). Furthermore, SynGAP also broadly modulates dendritic spine morphogenesis, receptor trafficking, and cytoskeleton remodeling by regulating other small GTPases such as Rap, Rab5, and Rac1. In excitatory synapses, *SYNGAP1* specifically downregulates the Ras–Raf–MEK–ERK signaling pathway by inactivating Ras superfamily small GTPases. This regulation is crucial for maintaining synaptic function and structural homeostasis, and its loss of function may lead to overactivation of this pathway, thereby triggering neurodevelopmental disorder-related phenotypes such as intellectual disability and autism-like behaviors (29). Other studies indicate that Cyfip1 regulates the synaptic expression of AMPARs, SynGAP1, and mGluRs. Reduced level of Cyfip1 enhances Ras signaling and downstream SynGAP1 activity, altering the balance between Ras and Rap signaling, ultimately changing the range and flexibility of synaptic responses (30).

### 4.3 Excitatory/inhibitory balance disruption and neural circuit abnormalities

The pathology of SRDs is not limited to excitatory neurons. Research has found that SynGAP is also expressed in inhibitory interneurons, particularly parvalbumin-positive (PV+) and somatostatin-positive (SST+) neurons, and plays a key role in their early development (31). *Syngap1* haploinsufficiency leads to specific impairments in these neurons. In PV + neurons, deficits include a reduced density of glutamatergic synaptic inputs on their somata, weakened spontaneous excitatory postsynaptic currents (sEPSCs), and decreased intrinsic excitability (increased action potential threshold, reduced firing frequency). These deficits are associated with abnormal regulation of Kv1 family D-type potassium currents (31). Zebrafish *syngap1b* knockdown models show a reduced number of GABAergic neurons in the midbrain and hindbrain regions, leading to an imbalance in the ratio of excitatory to inhibitory neurons, which may be related to seizure-like hyperexcitable behaviors and ineffective swimming locomotion (32).

Abnormal neural network activity is an important manifestation of *Syngap1* deficiency. Restricted knockdown of *Syngap1* in MGE-derived Nkx2.1-positive inhibitory interneurons leads to increased baseline gamma-band power in the adult mouse cortex, along with cognitive and social deficits, such as reduced social preference and impaired fear memory extinction. These neural network abnormalities are consistent with the regulatory role of *SYNGAP1* in brain regions like the cortex and hippocampus (33). Additionally, *Syngap1^+/−^* mice exhibit increased baseline *γ* oscillation power and enhanced *θ*/γ phase-amplitude coupling during wakefulness, lack adaptation to repetitive auditory stimuli, and have abnormal deviant sound detection. These sensory processing abnormalities align with clinical features of SRDs and can serve as translational biomarkers (34). The functional regulation of different types of MGE-derived inhibitory neurons by *Syngap1* is cell type-specific. This impaired function of inhibitory circuits directly leads to a severe imbalance between excitatory/Inhibitory (E/I) in the brain network, forming the common pathological basis for cognitive deficits, ASD-like behaviors, and epilepsy.

The impact of *SYNGAP1* deficiency on specific neuronal subtypes and their circuit functions is the core mechanism underlying the occurrence of cognitive and behavioral abnormalities. At the neuronal subtype level, *SYNGAP1* is primarily expressed in in cortical and forebrain glutamatergic neurons, where the *α* isoform (especially *α*1) is crucial for regulating synaptic function (10). Autonomous expression of *Syngap1* in cortical excitatory neurons is necessary for the assembly of the sensorimotor integration (SMI)-mediated perceptual network, and its loss disrupts the dynamics and structural function of the tactile perception-related cortico-thalamic circuit (35). In terms of neural circuit function, *SYNGAP1* deficiency causes multi-level abnormalities in synaptic connectivity and neural dynamics. On the one hand, loss of function of the *α*1 isoform, which localizes to the postsynaptic density via its PDZ-binding motif, reduces *Syngap1* content in the PSD, enhances excitatory synaptic function, and impairs long-term potentiation (LTP) (10). On the other hand, *Syngap1* mutant mice exhibit circuit-specific long-range synaptic connection abnormalities, such as hyperconnectivity from the motor cortex to layer 5 of the somatosensory cortex, while function of perception-related thalamic inputs is impaired, leading to dysregulated cortical sensory circuits (36).

### 4.4 Transcriptional and splicing regulatory mechanisms

Alternative splicing (AS) is a key mechanism for mRNA post-transcriptional gene diversification and regulation, particularly prevalent in the brain. The alternative splicing of neuronal genes is coordinately regulated by various RNA-binding proteins (RBPs), among which the polypyrimidine tract-binding proteins PTBP1 and PTBP2 play important roles. As a neuron-specific splicing regulator, PTBP2 binds to pre-mRNA targets, typically repressing inclusion of cassette exons when bound to canonical intronic polypyrimidine tracts upstream of these exons. CLIP-seq and splicing analysis have identified *SYNGAP1* as a direct target of PTBP2. PTBP2 binding to *SYNGAP1* pre-mRNA regulates its alternative splicing, specifically manifested as the selection of an alternative 3′ splice site for exon 11, introducing a premature termination codon, triggering nonsense-mediated decay (NMD), thereby limiting SYNGAP1 expression in neurons (37).

Alternative splicing of *SYNGAP1* not only affects its expression level but may also contribute to functional regulation through the generation of different isoforms. For example, in *Caenorhabditis elegans*, mutation of the ortholog gap-2/*SYNGAP1* increases neurite length and branch points in DVB neurons, suggesting a role in restricting experience-dependent neurite outgrowth that may be related to the functional diversity of splice isoforms (38). Furthermore, *SYNGAP1* participates in neurodevelopment by regulating excitatory/inhibitory (E/I) balance, and its function may depend on the expression of specific splice isoforms (39).

### 4.5 Protein homeostasis and epigenetic regulation

Chaperone-assisted protein folding is a primary mechanism for maintaining protein homeostasis. Among chaperones, HSP90 plays a central role in this process and is crucial in AMPA receptor synaptic transmission (40). SynGAP stability is precisely regulated. The Necdin protein stabilizes SynGAP by binding to the SGT1-HSP90 molecular chaperone complex, preventing its degradation (36). In the absence of Necdin, SynGAP protein levels are significantly decreased in the mouse brain, subsequently affecting dendritic spine maturation and synaptic transmission efficiency. This reveals a new pathological regulatory axis and offers a potential therapeutic target. Additionally, *SYNGAP1* also coordinately regulates synaptic function through epigenetic mechanisms. For example, *Syngap1^+/−^* mice show reduced p300/CBP histone acetylation. Activation of its KAT activity can restore synaptic plasticity, indicating synergy between structural function and epigenetic regulation (41).

## 5 Clinical phenotypes and construction of animal and cell models

### 5.1 Core clinical manifestations

All patients with SRDs exhibit varying degrees of developmental delay/intellectual disability, the majority of children (96%) already exhibited developmental delay prior to the seizures onset (4). Epilepsy is highly prevalent (69.6–98%), with diverse seizure types, including absence seizures, myoclonic seizures, eyelid myoclonia (often with absences), and atonic seizures (drop attacks), among others (generalized tonic–clonic seizures, focal seizures, spasms) (4, 42–44). A relatively characteristic manifestation is reflex epilepsy, particularly triggered by eating/chewing (45). Electroencephalography (EEG) often shows generalized or posterior-dominant epileptiform discharges, which are enhanced during sleep. EEG findings may include an ECS pattern (manifesting as rhythmic posterior/diffuse *δ* waves upon eye closure that persist into the eye-open state) and an FOS pattern (characterized by diffuse polyspike discharges triggered by eye closure, specifically in cases of visually sensitive epilepsy) (44). Common comorbidities include ASD, aggressive/impulsive behaviors, sleep disorders, and high pain threshold, feeding difficulties, and ataxia or gait abnormalities (4, 46, 47).

### 5.2 Construction and phenotypes of animal and cell models

Mouse models are the most widely used experimental systems for studying *SYNGAP1* loss of function, including heterozygous knockout, conditional knockout, and knock-in models carrying human pathogenic variants. *Syngap1^+/−^* heterozygous mice show a reduction in cortical SynGAP1 protein expression to 41% of wild-type levels (1), while knock-in mice carrying human SRDs pathogenic mutations (such as the L813RfsX22 frameshift mutation and the c.3583-9G > A intronic splicing mutation) exhibit 30–50% reduction in *Syngap1* mRNA and approximately 50% reduction in SynGAP1 protein expression, validating haploinsufficiency as the core mechanism of SRDs pathogenesis (48).

In terms of behavioral phenotypes, *Syngap1* deficient mice display various abnormalities. Common features include hyperlocomotion, impaired working and reference memory, and deficits in social behavior, such as reduced social interaction time and impaired preference for social novelty (3). Regarding emotion and sensation-related behaviors, *Syngap1^+/−^* and *Syngap1^−/+^* mice both show reduced anxiety-like behaviors in the open field and elevated plus maze tests. *Syngap1^−/+^* mice also exhibit reduced sensitivity to painful stimuli in the hot plate test (3) and display abnormal risk-taking behaviors, such as increased time in the open arms of the elevated plus maze and more frequent departures from the safe platform in the cliff avoidance test (49).

*Syngap1* Het mice exhibit bilateral generalized sharp wave discharges on EEG, suggesting neural circuit hyperexcitability. Sleep architecture is also disrupted. *Syngap1^+/−^* mice show increased active wake time, decreased quiet wake and slow-wave sleep time, and exacerbated high-amplitude intermittent discharges (IIS) during sleep (28), a phenomenon similarly observed in *SYNGAP1* patients. Furthermore, *Syngap1* deficient mice show attenuated hippocampal *θ* oscillations. Notably, specific re-expression of SynGAP protein in adulthood significantly increases θ oscillation amplitude, improving cognitive function and neural excitability (50). Importantly, even at older ages (over 53 weeks) on the JAX C57BL/6 J genetic background, *Syngap1^−/+^* mice, stably recapitulate the major neurobehavioral phenotypes of patients with *SYNGAP1*-related intellectual disability and autism spectrum disorder, including motor, cognitive, social, and sensory processing abnormalities (51).

### 5.3 Value of animal models

Genetically engineered mouse models (e.g., heterozygous knockout, knock-in models carrying human pathogenic mutations) are core tools for investigating SRDs mechanisms. These models successfully recapitulate core clinical phenotypes, including hyperactivity, cognitive memory deficits, social abnormalities, epileptiform discharges, and abnormal neural oscillations (1, 48). Cell type-specific knockout models have revealed the differential functions of SynGAP in excitatory neurons and specific types of inhibitory neurons. Additionally, zebrafish models, with their transparency and suitability for whole-brain imaging, play a role in behavior and pharmacological screening studies at the neural network level (52).

However, current animal models have significant limitations. Traditional whole-gene knockout models cannot effectively distinguish the independent contributions of SynGAP’s enzymatic (GAP) activity versus its structural scaffolding function to specific phenotypes (25, 27). Most models carry complete loss-of-function alleles, which may not accurately reflect the spectrum of human mutations (e.g., missense variants affecting specific domains). Species differences in neural circuit complexity, developmental timelines, and drug metabolism also pose challenges for direct translation to humans (53). Patient-derived induced pluripotent stem cell (iPSC) models offer a complementary approach to study human neuronal development and mutation-specific effects in a controlled genetic background (39).

## 6 Treatment strategies and progress

### 6.1 Traditional anti-seizure medications and symptomatic treatment

Pharmacological treatment of SRDs relies primarily on retrospective clinical data. Large-scale retrospective studies indicate that valproate and lamotrigine show relatively favorable efficacy in controlling seizures, while levetiracetam is less effective (47). Behavioral comorbidities are often addressed with medications such as risperidone, aripiprazole, and guanfacine. Currently, no curative therapy exists for *SRDs*. Pharmacological treatment primarily targets symptoms like epilepsy and sleep disorders, yet significant cognitive and motor impairments persist. Consequently, behavioral interventions and rehabilitation therapies (e.g., physical, occupational, speech therapy) play an important auxiliary role in improving quality of life for patients and functional outcomes (54).

### 6.2 Potential beneficial molecularly targeted drugs

AMPA receptor antagonists: Based on the mechanism of AMPAR hyperfunction in SRDs, low doses of the AMPAR antagonist perampanel show therapeutic potential in animal models and case reports. Studies confirm that it reverses cortical gamma oscillation abnormalities in *Syngap1^+/−^* mice (55). In a 25-month-old SRDs child, low-dose perampanel not only improved sleep-stage gamma wave disruption but also enhanced sleep quality and developmental miletones (such as walking, communication), suggesting its potential disease-modifying effects (56). However, larger controlled clinical trials are needed to confirm its efficacy and optimal dosing, especially for cognitive benefits.

Statins (e.g., Lovastatin) exert neuroprotective effects through pleiotropic mechanisms. Studies show they downregulate the NMDA receptor NR2B subunit, reduce excitotoxicity, and negatively regulate the Ras–ERK signaling pathway by inhibiting prenylation. In a chronic hypoxic hypercapnic rat model, lovastatin improved spatial learning and memory, accompanied by upregulated levels of pERK1/2, pCREB, and BDNF in the hippocampus (57). Clinical case reports confirm that low-dose lovastatin (20 mg/day) improve behavioral symptoms in a patient with *SYNGAP1* mutation, although efficacy is dose-dependent, and higher doses may cause adverse effects (58). Rosuvastatin also shows potential for reducing seizures in a patient with *SYNGAP1*-related epilepsy (59). In summary, statins are a potential therapeutic strategy for cognitive and behavioral disorders, though further research is needed to clarify their efficacy, optimal dosage and long-term safety profile in SRDs.

### 6.3 Precision gene and molecular therapy

This is currently the most promising research direction, aiming to restore SynGAP protein expression and function at the molecular level.

#### 6.3.1 Gene supplementation and gene editing therapy

Adeno-associated virus (AAV) vector-based *SYNGAP1* gene supplementation strategies are an important current research direction. Researchers have developed a strategy using the pan-neuronal promoter *SYNAPSIN* I to drive widespread expression of the full-length human *SYNGAP1* gene in neurons. Their AAV vector packages the transgene encoding the *SYNGAP1-α1* isoform. In the Syngap1 heterozygous mouse model, this gene supplementation approach partially rescued epileptiform activity and selected behavioral phenotypes in mouse models. At the electrophysiological level, it not only significantly reduced the frequency of interictal spike waves but also improved abnormalities in multiple frequency bands of the EEG power spectrum, achieving normalization of neural oscillations. This study was the first to demonstrate that an AAV-mediated full-length *SYNGAP1* gene supplementation strategy not only restores *SYNGAP1* expression biochemically but also reverses key phenotypes of *SRDs* at the behavioral and electrophysiological functional levels (60). This strategy is particularly suitable for intervention during the early developmental window. Key challenges for clinical translation include optimizing AAV serotypes and delivery routes for efficient and safe brain-wide transduction in humans, potential immune responses, and determining the optimal therapeutic time window.

#### 6.3.2 Antisense oligonucleotide (ASO)-mediated splicing modulation

Antisense oligonucleotide (ASO)-mediated splicing modulation is a therapeutic strategy that targets the alternative splicing process of *SYNGAP1* pre-mRNA to increase the expression of functional SynGAP1 protein. Its core mechanism involves interfering with the inhibitory effect of RNA-binding proteins on *SYNGAP1* splicing, thereby redirecting the splicing pattern, reducing the generation of non-productive transcripts, and promoting the production of mature mRNA. Studies show that PTBP family proteins (including PTBP1 and PTBP2) play a key role in *SYNGAP1* splicing regulation. They bind to specific regions of *SYNGAP1* mRNA, promoting non-productive alternative splicing events and subsequent nonsense-mediated decay (NMD), leading to limited *SYNGAP1* expression (37). Based on this mechanism, designing ASOs targeting PTBP binding sites can interfere with the PTBP-*SYNGAP1* mRNA interaction, relieving splicing repression, redirecting the splicing pattern, and thereby increasing functional *SYNGAP1* mRNA and protein levels in patient-derived iPSC neurons and mouse models. This strategy has been shown to increase SYNGAP1 mRNA and protein expression in human and patient-derived iPSC neuronal models (61). PTBP2 knockdown or ASO treatment can partially restore SYNGAP1 expression levels in selected experimental systems. While the preclinical rationale is compelling, it is important to emphasize that these efficacy data are derived from animal models and *in vitro* systems.

In therapeutic research for *SRDs*, optimization of drug delivery systems is crucial for improving the brain distribution of therapeutic agents and enhancing efficacy, with blood–brain barrier (BBB) penetration being a key challenge. ASOs, as a promising therapeutic, have their brain delivery efficiency directly affecting treatment outcomes. In the aforementioned study (37), the researchers used intracerebroventricular injection to deliver ASOs to neonatal mouse brains to validate *in vivo* effects. This delivery strategy directly introduces the drug into the cerebrospinal fluid, bypassing the blood–brain barrier’s restriction on large molecule drugs, allowing ASOs to distribute more effectively in brain tissue, ultimately significantly upregulating *Syngap1* mRNA expression. CAMP4 Therapeutics is developing an ASO drug (CMP-SYNGAP-01) based on this principle. Company-reported preclinical data suggest increased SynGAP1 protein levels and behavioral effects in animal models; however, these findings have not yet been independently validated. Broader challenges for ASO therapy include efficient and sustained delivery across the BBB in humans, potential off-target effects, and the applicability of splicing modulation across diverse SYNGAP1 mutation types.

#### 6.3.3 Translation-activating RNAs (taRNAs)

Emerging translation-activating RNA technology provides a new therapeutic strategy for *SRDs*. Translation-activating RNAs (taRNAs) are bifunctional RNA molecules that specifically bind to target mRNA and directly promote its translation. taRNAs consist of a guide sequence and an effector domain selected from viral or mammalian internal ribosome entry sites (IRES), which enhance protein synthesis of the target mRNA by recruiting translation initiation factors eIF3 and eIF4G. Through structural optimization, researchers have developed a minimized taRNA to 94 nucleotides that achieved effective activation of SYNGAP1 expression. In terms of delivery, taRNAs can be delivered via lipid nanoparticles (LNPs) to cell lines, primary neurons, and *in vivo* in mice. This approach represents a potential non-viral delivery strategy that warrants further evaluation in preclinical models. In patient-derived cell models, taRNAs successfully amplified SYNGAP1 expression in patient-derived cellular models, resulting in increased SynGAP protein levels (62). This approach is in early preclinical development. Major hurdles include achieving efficient, specific, and durable in vivo delivery to the central nervous system with LNPs, minimizing immune activation, and demonstrating functional rescue in animal models of SRDs, and ultimately addressing the scalability and safety manufacturing of complex RNA-LNP formulations for chronic neurological conditions.

## 7 Challenges and future directions

Despite significant progress, research and therapy for SRDs still face multiple challenges:

### 7.1 SynGAP1 protein isoform functions and their pathological relevance

The *SYNGAP1* gene produces a rich variety of protein isoforms through multiple transcription start sites and alternative splicing mechanisms, including N-terminal A, B, C isoforms and C-terminal *α*1, *α*2, *β*, *γ* isoforms, among others. Most functional differences stem from the C-terminal region, and at least 12 different protein isoforms have been identified, indicating complex functional diversity. These isoforms exhibit significant heterogeneity in spatial and temporal expression, which may be an important basis for the clinical symptom heterogeneity in *SRDs*. In terms of temporal expression characteristics, different isoforms show distinct development-dependent expression patterns. For example, the *α*1 isoform expression starts later in the cortex and hippocampus, with low levels in early brain development (first two postnatal weeks), but increases substantially in the cortex and hippocampus thereafter, remaining enriched in the postsynaptic density (PSD) in later stages. This suggests that early brain development may primarily rely on other isoforms like *α*2 and *β* (8). Currently, the precise functions of the various SynGAP splice isoforms in specific brain regions, cell types, and developmental stages remain unclear, complicating the selection of optimal therapeutic targets (e.g., which isoform to supplement).

### 7.2 Unresolved issues in pathological mechanisms and model refinement

Current research on the pathological mechanisms of *SRDs* still faces many unresolved issues, mainly reflected in the systematic limitations of existing research models and the complexity of parsing the pathological network. In animal models, traditional *Syngap1* whole-gene knockout models have the defect of mixed functions, unable to effectively distinguish the independent roles of different functional domains of the SynGAP1 protein (e.g., enzymatic activity vs. structural function). To address these shortcomings, model systems require refinement along several dimensions: first, developing knock-in models that accurately mimic specific mutations in human patients; second, constructing mutation models that distinguish between different functional domains of SynGAP1; third, expanding cross-species models (e.g., zebrafish), combined with whole-brain functional imaging techniques to analyze neural circuit dynamics (53). Additionally, the diversity of intrinsic metabolic and biochemical pathways in different animal strains and species leads to variations in pharmacokinetics and pharmacodynamics of drugs within the system and shows differences from humans. A feasible alternative to overcome these problems is the use of patient-derived induced pluripotent stem cells (39). Simultaneously, the integration of multi-omics technologies is key to parsing complex pathological mechanisms, requiring the combination of single-cell sequencing, spatial transcriptomics, proteomics with neuroelectrophysiology, and structural/functional connectivity analysis to achieve multi-scale mechanistic analysis from molecular, cellular to circuit levels. The same gene mutation can lead to different clinical phenotypes. Furthermore, different mutations types (e.g., truncating vs. missense) have varying impacts on protein function (enzyme activity vs. structural function). This complexity requires more refined analysis of genotype–phenotype correlations (20, 21).

### 7.3 Optimization of treatment strategies

Delivery efficiency and safety: Key challenges include the efficient and safe intracranial delivery of large molecule drugs such as AAV and ASOs. Future efforts will need to focus on optimizing delivery systems (e.g., novel AAV serotypes, nanocarriers) and administration routes is a future priority.

Therapeutic Window: Brain development has critical periods, making the timing of intervention crucial. Although re-expressing SynGAP in adulthood can improve some functions, early intervention may be more effective (63).

Personalized Treatment: In the future, it may be necessary to select the most appropriate treatment strategy based on the patient’s specific mutation types and clinical phenotypes, such as using ASO for patients with splicing mutations and gene supplementation for those with truncating mutations.

### 7.4 Clinical trial design and biomarkers

The core challenges in clinical trials of SRDs include phenotypic heterogeneity, a lack of objective biomarkers, and limitations of response assessment tools. The clinical heterogeneity of SRDs complicates the selection of endpoints for clinical trials. Recent research on quantitative electroencephalogram (qEEG) biomarkers in genetic epilepsies and their relationship to neurodevelopmental outcomes indicates that patients with SRDs primarily exhibit the following qEEG characteristics: spectral abnormalities in the occipital region; a reduced *α*-δratio, particularly in early childhood, which can serve as a key distinguishing feature. Furthermore, EEG spectral features are associated with motor development—a phenomenon not exclusive to patients with genetic epilepsies—but do not predict seizure control (64). Patients with SRDs often present with sensory processing abnormalities. Studies on Syngap1−/+ mouse models suggest that these sensory processing deficits may be related to reduced cortical synaptic connectivity (37). Research by Lyons-Warren AM et al. demonstrates that patients with SRDs score higher in the avoiding and seeking quadrants, particularly in the avoiding quadrant. Additionally, a correlation exists between sensitivity and registration (65). Developing reliable biomarkers (e.g., gamma oscillation abnormalities in EEG, sensory processing indicators) is crucial for objectively assessing efficacy (34, 63).

McKee et al. (47) demonstrated a more than fivefold increased risk of autistic behaviors occurring between 27 and 30 months of age, with a significant increase in generalized seizures after 3 years of age. Therefore, a multi-dimensional efficacy evaluation system is also essential. Disease concept models established jointly by patient families and clinical experts indicate that future trials need to comprehensively assess multiple aspects such as seizure frequency, cognitive function, emotional behavior, and communication ability, balancing medical objective indicators with patient-reported outcomes. Building large-scale, standardized patient registry systems and natural history databases is vital. International collaborative projects, such as the “*SYNGAP1* Census” led by the *SYNGAP1* Research Fund (SRF) (54) and the US-based Brain Gene Registry (BGR) (9), are systematically collecting genomic, deep phenotypic, and real-world data. These efforts aim to precisely delineate the full clinical spectrum of SRDs, clarify its developmental trajectory, and identify suitable biomarkers, efficacy endpoints, and intervention time windows for clinical trials, providing historical control data for randomized controlled trials and forming the basis for designing and successfully executing clinical trials.

### 7.5 Prospects for precision therapy and personalized treatment

Precision therapeutic strategies based on molecular pathological mechanisms need to target SynGAP protein homeostasis regulation and structural function. The “Necdin-SGT1-HSP90” axis, as a key regulatory pathway for maintaining SynGAP protein stability, whose dysfunction can lead to reduced protein levels and synaptic transmission imbalance, suggests a promising target for precise intervention (36). Given the multi-domain nature of SynGAP, combination strategies that synergistically target chaperone-mediated protein stabilization, PSD site occupancy regulation, TARP-AMPAR complex dynamic balance, and CaMKII-related signaling pathways may achieve comprehensive regulation of synaptic transmission and neurodevelopment. Regarding early intervention, given SynGAP’s important role during critical periods of neurodevelopment, combining technologies like taRNAs that can be efficiently delivered and precisely upregulate endogenous proteins, along with early regulation of pathways like the “Necdin-SGT1-HSP90” axis, holds promise for reversing synaptic abnormalities and behavioral deficits early in the disease process. The ultimate goal is a personalized medicine framework where treatment is tailored to the individual’s mutation type, age, and dominant clinical features, informed by robust biomarkers and a deep understanding of natural history data and a deep understanding of the underlying molecular lesion. This vision, however, is contingent upon overcoming the significant translational challenges outlined above.

## 8 Conclusion

*SYNGAP1*-related neurodevelopmental disorder (SRD) is a severe brain disease caused by haploinsufficiency of a core synaptic regulatory protein. Its pathogenesis involves multi-level defects, from molecular signaling pathway disruption to cellular dysfunction, and further to neural circuit imbalance. Recent research has not only deeply revealed the dual roles of SynGAP in GAP activity and structural function and its central position in excitatory/Inhibitory (E/I) balance but has also catalyzed the development of multiple highly promising precision therapeutic strategies.

From traditional anti-seizure medications to cutting-edge gene supplementation, ASO splicing modulation, and taRNA translation activation, the treatment paradigm is undergoing a fundamental shift. Future research should focus on elucidating protein isoform functions, optimizing delivery technologies, defining the optimal therapeutic window, and, through interdisciplinary collaboration and deep engagement with patient communities, promote the translation of these innovative therapies from the laboratory to the clinic, ultimately bringing hope and substantial life improvements to patients with SRDs.
